# Reviving Consciousness: A Neurophysiotherapy Triumph in Decompressive Craniotomy Recovery

**DOI:** 10.7759/cureus.52278

**Published:** 2024-01-14

**Authors:** Anandi R Dave, Nikita H Seth, Snehal Samal

**Affiliations:** 1 Neuro Physiotherapy, Ravi Nair Physiotherapy College, Datta Meghe Institute of Higher Education and Research, Wardha, IND

**Keywords:** postoperative cognitive dysfunction, neurophysiotherapy rehabilitation, multimodal stimulation, hemmorhagic stroke, decompressive craniotomy

## Abstract

This case report presents a 54-year-old male with a history of type-2 diabetes mellitus who experienced sudden unconsciousness and vomiting, leading to aspiration and subsequent diagnosis of a hemorrhagic stroke. The patient underwent an immediate decompressive craniotomy, revealing a sizable intraparenchymal hematoma in the right basal ganglia and corona radiata. Postoperatively, the patient exhibited left-sided weakness, hyporeflexia, and cognitive impairment. A comprehensive neurophysiotherapy intervention addressed impaired mobility, strength, balance, coordination, respiratory complications, pain management, and other associated challenges. The rehabilitation protocol involved diverse strategies such as passive and active exercises, sensory stimulation, and the application of neurophysiotherapeutic approaches. The patient's progress was assessed using various outcome measures. Neurophysiotherapy plays a crucial role in the recovery of decompressive craniotomy.

## Introduction

Hemorrhagic stroke, a critical neurological event characterized by bleeding within the brain, represents a profound clinical challenge necessitating comprehensive management strategies [1]. This case report centers on the rehabilitation journey of an individual who underwent a decompressive craniotomy following a hemorrhagic stroke, with a specific focus on the integral role played by neurophysiotherapy in optimizing recovery. Hemorrhagic strokes arise from the rupture of blood vessels within the brain, leading to bleeding in the surrounding tissues. Causes of hemorrhagic strokes encompass various factors, including hypertensive emergencies, cerebral aneurysms, arteriovenous malformations, and anticoagulant medication usage [2]. The clinical consequences of hemorrhagic strokes are severe, often resulting in neurological deficits and impaired functional abilities. Management strategies involve urgent medical interventions, including surgical procedures such as decompressive craniotomy, aimed at alleviating intracranial pressure and preventing further damage [3]. Decompressive craniotomy emerges as a critical surgical intervention in the context of hemorrhagic strokes. Indicated when intracranial pressure becomes dangerously elevated, this procedure involves the removal of a portion of the skull to allow for the expansion of the brain, mitigating the risk of life-threatening complications. The decision for decompressive craniotomy is influenced by factors such as the extent of hemorrhage, mass effect, and neurological status [4].

The occurrence of aspiration pneumonia, a condition characterized by inflammation of the lungs due to the inhalation of foreign material, adds a layer of complexity to the aftermath of a hemorrhagic stroke [5]. Aspiration events are particularly relevant in stroke cases, where impaired swallowing mechanisms can lead to the inhalation of oral or gastric contents. Aspiration pneumonia is a severe complication, often requiring prompt attention and targeted interventions to prevent respiratory compromise and enhance overall recovery [6]. Neurophysiotherapy plays a pivotal role in the rehabilitation process following a decompressive craniotomy for hemorrhagic stroke [7,8]. The multifaceted approach addresses impairments in mobility, strength, balance, coordination, respiratory function, and cognitive abilities. The physiotherapy protocol includes tailored interventions such as passive and active range of motion (ROM) exercises, balance training, respiratory exercises, cognitive rehabilitation, and sensory-motor approaches like Rood's techniques, multimodal stimulation, and Proprioceptive Neuromuscular Facilitation (PNF) [9-15].

Through this case report, we aim to delineate the intricate relationship between hemorrhagic strokes, decompressive craniotomy, aspiration pneumonia, and the indispensable role of neurophysiotherapy in optimizing the rehabilitation process [16]. The subsequent sections will provide a detailed exploration of the patient's clinical course, the rationale behind neurophysiotherapy interventions, and the collective effort to enhance functional outcomes and quality of life (QOL).

## Case presentation

The subject of this case presentation is a 54-year-old male known for type-2 diabetes mellitus for 15 years and was on regular medication for the same. He had right-hand dominance and experienced sudden unconsciousness and vomiting in his sleep, which got aspirated in September 2023. After this, the patient got unconscious and was immediately taken to a local hospital. Due to unavailability, the patient did not get treatment and was taken for investigation the following day. Studies such as computed tomography (CT) scans of the brain revealed hemorrhagic stroke, with intraparenchymal hematoma of 72x35 mm in the right basal ganglia and corona radiata. A mass is noted over the right lateral ventricle and a midline shift of 7 mm to the left side. Biochemistry revealed increased serum cholesterol (210 mg/dl), increased high-density lipoprotein (HDL) (5.5), increased blood urea (65.1 mg/dl), and increased serum creatinine (1.58 mg/dl).

The patient was operated immediately on the same day. The patient gained consciousness in three days but was not oriented. Chest CT revealed airspace consolidation in bilateral lower limbs, mild bilateral pleural effusion, and aspiration pneumonitis. The patient’s relatives complained of an inability to move the left side of the body. As mentioned in Table 1, the reflexes of joints were assessed. Hyporeflexia was noted in the left upper and lower extremities. The left upper and lower limb tones were seen as a two-layer in Table 2 using the tone grading scale.

**Table 1:** Joint reflexes Ab: Absent

Reflexes	Right	Left
Biceps reflex	++	Ab
Triceps reflex	++	+
Brachioradialis reflex	++	Ab
Patellar reflex	++	Ab
Achilles reflex	++	Ab
Plantar reflex	Flexor	Flexor

**Table 2:** Tone grading scale TGS: Tone Grading Scale, 0: No response, 1+: Decreased response, 2+: Normal response, 3+: Exaggerated response, 4+: Sustained response

Joints	TGS (Right)	TGS (Left)
Shoulder flexors	2	1
Shoulder extensors	2	1
Elbow flexors	2	1
Elbow extensors	2	1
Wrist flexors	2	1
Wrist extensors	2	1
Hip flexors	2	1
Hip extensors	2	1
Knee flexors	2	1
Knee extensors	2	1
Ankle plantar flexors	2	1
Ankle dorsiflexors	2	1

Investigations

Figure 2 shows evidence of extra-axial blood density collection (Hounsfield unit (HU) +50 to +90) in the posterior falx and left tentorium cerebelli suggestive of subdural hemorrhage. There was evidence of well-defined intraaxial blood density collection (HU +50 to +80) in the left temporo-parieto-frontal lobe measuring approximately 8.4 x 5.6 x 3.4 cm with perilesional edema extending into the body of left lateral ventricle suggestive of hemorrhagic contusion with intraventricular extension.

**Figure 1:**
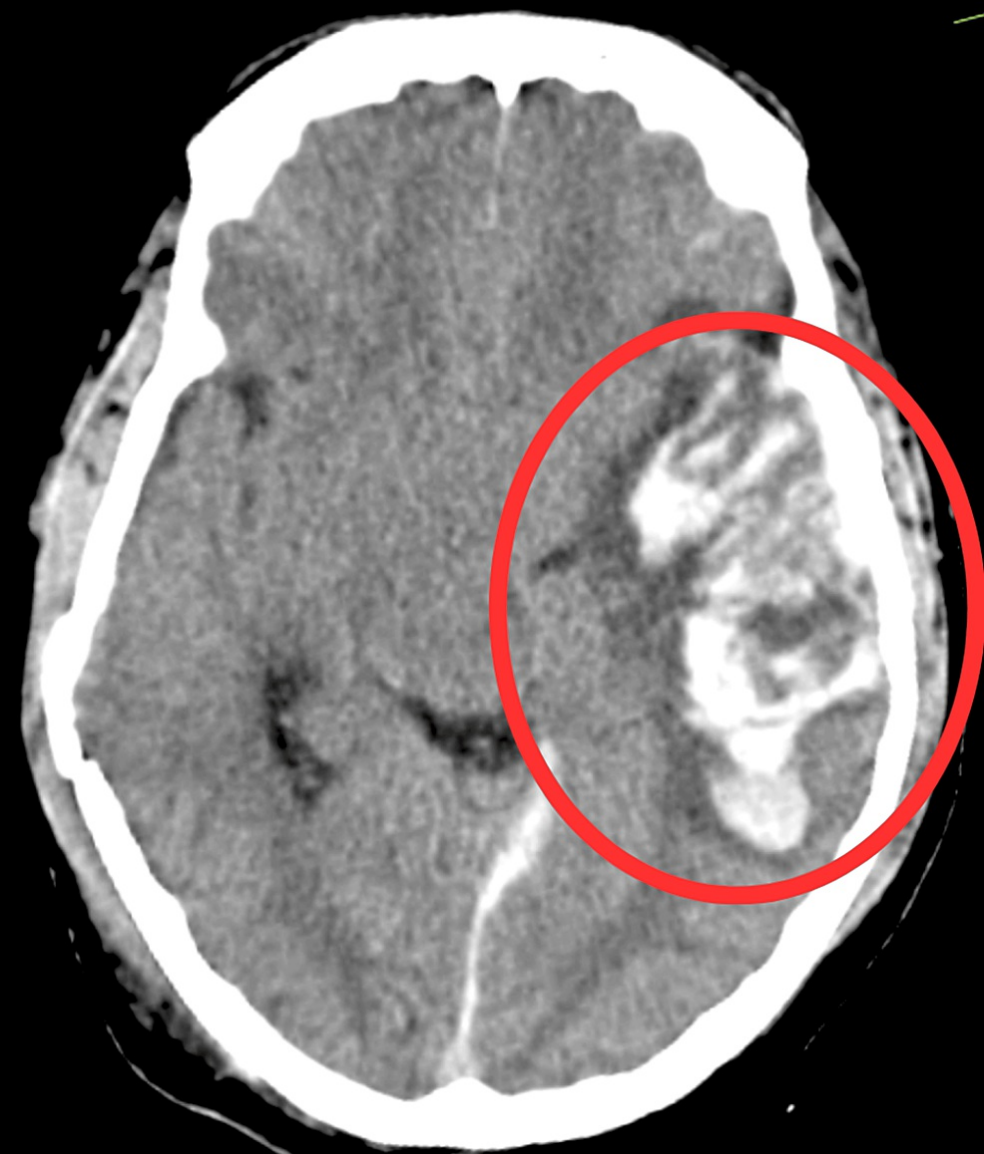
Pre-operative computed tomography scan The red oval shows hemorrhagic contusion in the left temporo-parieto-frontal region with intraventricular extension and mass effect

Physiotherapy intervention

**Figure 2:**
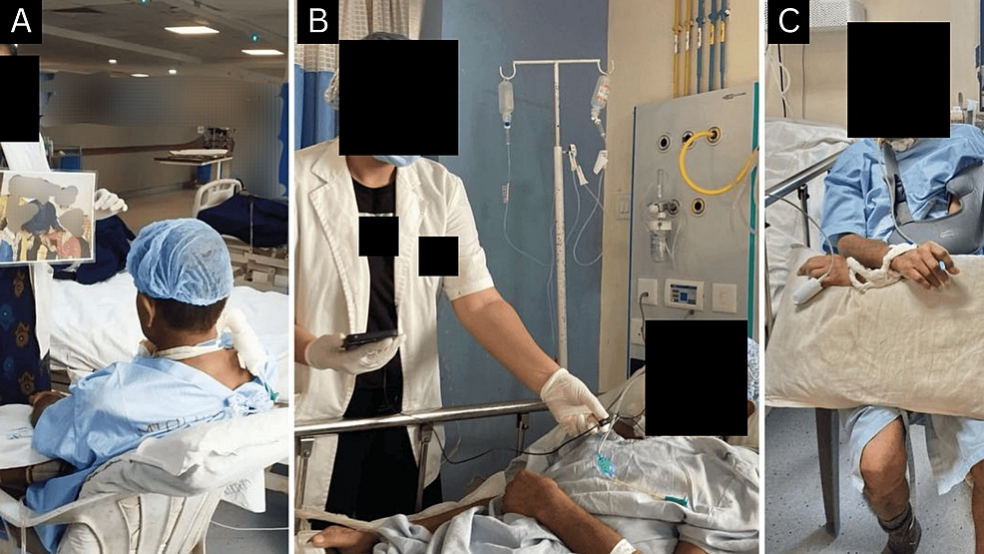
Physical therapy rehabilitation A: Multimodal stimulation (visual); B: Multimodal stimulation (auditory); C: Sitting on a chair at the bedside.

**Figure 3:**
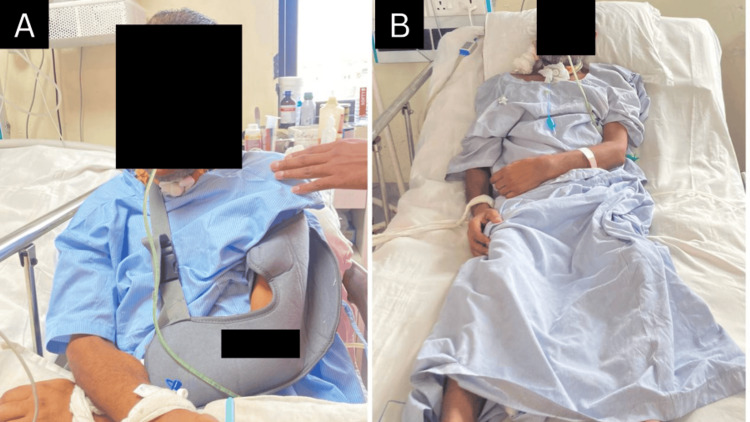
Rehabilitation A: Patient wearing shoulder arm pouch; B: Patient is in supine with head end elevated to 45 degrees

The patient was given physiotherapy rehabilitation seven days a week for one hour daily, for two months. The protocol to be followed using a timeline of the rehabilitation is presented in Table 3. The patient being treated is shown in Figures 2, 3.

**Table 3:** Physiotherapy rehabilitation intervention ROM: Range Of Motion; TENS: Transcutaneous Electrical Nerve Stimulation; PNF: Proprioceptive Neuromuscular Facilitation, QOL: Quality of Life

Sr. no.	Problem	Goal	Intervention	Rationale
1.	Unawareness about the condition	Increase awareness and education about the medical condition	Family education sessions encourage active participation in treatment decisions	Promotes understanding of the condition and the importance of rehabilitation, empowers the patient and encourages active involvement in the recovery process
2.	Multimodal stimulation	Optimize neuroplasticity and functional recovery	Visual stimulation leading visual tracking, tactile stimulation	Enhance neuroplasticity, improve sensorimotor integration, and facilitate recovery through diverse sensory inputs.
3.	Cognitive impairment (cognitive impairment assessed through RLA scale)	Enhance cognitive function	Cognitive exercises focusing on long term memory, attention, and problem solving, environmental modifications for safety and orientation	Cognitive exercises focusing on memory, attention, and problem-solving, environmental modifications for safety and orientation
4.	Respiratory complications	Improve respiratory function	Breathing exercises like diaphragmatic breathing; incentive spirometry, chest physiotherapy (10 repitions x 1 set)	Aids in lung expansion and ventilation, helps prevent respiratory complications
5.	Prone to develop bed sores	Prevent pressure ulcers and promote skin integrity	Bed mobility and positioning strategies (20 mins x 4 times/ day)	Regular repositioning and offloading techniques, skin inspections and skincare routines, education on proper nutrition and hydration
6.	Risk of contractures	Prevent joint contractures and maintain joint mobility	Regular positioning, passive ROM exercises, splinting	Counteracts the effects of immobility and reduces the risk of joint stiffness, promotes optimal joint mobility
7.	Pain management	Alleviate pain and discomfort	Therapeutic modalities like IRR for bed sores, EMS for muscle re-education (30 contractions x 3 sets)	Provides relief from pain and discomfort Improves overall comfort and promotes relaxation
8.	Decreased ROM	Restore and maintain joint mobility	Passive ROM of affected side; active ROM of unaffected side; stretching and flexibility exercises	Prevents the development of joint contractures Improves flexibility and joint mobility
9.	Impaired mobility/ strength	Improve strength and restore functional mobility	Passive ROM exercises; functional mobility training	Enhances neuromuscular coordination and muscle strength, facilitates the relearning of normal gait patterns Improves independence in daily mobility tasks
10.	Swallowing difficulty	Enhances swallowing function and safety	Dysphagia therapy with swallowing exercises, modified diet and positioning recommendations	Strengthens swallowing muscles and improves coordination, reduces the risk of aspiration and ensures safe oral intake
11.	Neurological deficits (Rood’S Approach)	Facilitate motor responses using Rood’s techniques	Tapping, brushing, proprioceptive input	Activate sensory-motor pathways, facilitate motor responses, and enhance overall motor function.
12.	PNF	Improve neuromuscular coordination	PNF patterns, rhythmic exercises	Enhance neuromuscular coordination, improve muscle strength, and promote functional movement.
13.	Balance and coordination deficit	Enhance balance and coordination	Sitting static and progressed to dynamic balance exercises on swiss ball and mat, coordination drills incorporating various limb movements and patterns	Challenges and improves the patient's ability to maintain equilibrium, promotes sensorimotor integration and coordination
14.	Gait disturbances	Improve gait pattern	Gait training, use of assistive device	Enhance walking ability and safety, reduces the risk of falls
15.	Communication challenges	Improve communication skills	Speech therapy	Promotes understanding of the condition and the importance of rehabilitation, empowers the patient and encourages active involvement in the recovery process
16.	Functional independence	Enhance independence in daily activities	Task-specific activities tailored to individual needs and goals Adaptive equipment and techniques for self-care tasks	Facilitates the reintegration of the patient into daily life Improves overall QOL by promoting independence

Outcome measures

The patient was assessed during the initial week, and progress was measured during the fourth and eighth weeks of physiotherapy rehabilitation, as shown in Table 4.

**Table 4:** Outcome measures ICU: Intensive Care Unit; RASS: Richmond Agitation Sedation Scale; FIM: Functional Independence Measure; GCS: Glasgow Coma Scale; RLAS: Rancho Los Amigos level of cognitive functioning.

Outcome measures	Week 1	Week 4	Week 8
ICU mobility scale	0	1	3
RASS score	-4	-1	0
FIM	Level 1	Level 2	Level 4
GCS	3	6	10
Disability rating scale	0	2	3
Coma recovery scale	1	9	19
RLAS	Level 1	Level 5	Level 8

## Discussion

The study by Grüner et al. investigates the application of Multimodal Early Onset Stimulation (MEOS) in the early rehabilitation of severe brain injury patients. Highlighting the importance of early intervention, the study focuses on a specific patient selection criterion and outlines the multimodal components of MEOS, including acoustic, tactile, olfactory, gustatory, and kinesthetic procedures. Monitoring responses through physiological and behavioral parameters, the study identifies significant changes in vegetative functions, mainly influenced by tactile and acoustic stimulation. While acknowledging controversies in therapeutic effects, the authors emphasize the individualized nature of rehabilitation and the potential diagnostic value of non-responsiveness to external stimuli. The study contributes insights into sensory stimulation and calls for further research to refine protocols and prognostic assessments [17].

This article highlights the crucial role of neurorehabilitation in the aftermath of stroke, emphasizing its significance in reducing mortality, disability, and overall healthcare costs associated with this condition. The prevalence of stroke necessitates a comprehensive approach to stroke management, with a focus on acute neurological treatment, stroke unit admissions, fibrinolytic interventions for ischemic strokes, and, notably, neurorehabilitation. Given their expertise in neuroanatomy, physiopathology, neuropharmacology, and brain plasticity, Neurologists are positioned to actively contribute to neurorehabilitation teams. The article underscores vital factors influencing the efficacy of rehabilitation, emphasizing the importance of early initiation, optimal duration, and treatment intensity. It contends that a concerted effort is essential to ensure timely and appropriately intense neurorehabilitation during inpatient and outpatient phases, as this correlates with improved functional outcomes, reduced mortality and institutionalization rates, and shorter hospital stays. Ultimately, the article advocates for integrating neurologists into multidisciplinary neurorehabilitation teams and underscores the need for strategic planning to facilitate timely and sustained neurorehabilitation for stroke patients [18].

The study by Kelly et al. investigated functional recovery in intracerebral hemorrhage (ICH) and cerebral infarction patients undergoing rehabilitation. Analyzing over 1,000 cases, they found that, despite ICH patients having greater initial impairment, they experienced more significant gains during rehabilitation than cerebral infarction patients. Surprisingly, the initial severity of disability did not predict the degree of recovery. Instead, younger age, a longer length of stay, and cognitive function at admission were identified as predictors of better outcomes. The study challenges traditional assumptions and suggests tailoring rehabilitation strategies based on stroke subtypes and individual characteristics [19].

In summary, these studies underscore the evolving landscape of neurorehabilitation, emphasizing the need for personalized, timely, and intensive interventions. They contribute to the ongoing discourse on refining rehabilitation protocols, prognostic assessments, and the integration of neurologists into multidisciplinary teams, ultimately striving for improved outcomes and enhanced quality of life for patients recovering from severe brain injuries and strokes.

## Conclusions

This case underscores the critical role of neurophysiotherapy in the multidisciplinary management of a hemorrhagic stroke patient following decompressive craniotomy. The tailored rehabilitation protocol, incorporating elements of sensory stimulation and neurophysiotherapeutic approaches, demonstrated positive outcomes regarding motor function, cognition, and overall well-being. The findings emphasize the importance of early and individualized rehabilitation strategies to optimize recovery and functional independence in patients with hemorrhagic stroke undergoing decompressive craniotomy. Further research and larger-scale studies are warranted to refine and validate such rehabilitation protocols in diverse clinical settings.
